# Understanding the Pathophysiology of COVID-19: Could the Contact System Be the Key?

**DOI:** 10.3389/fimmu.2020.02014

**Published:** 2020-08-11

**Authors:** Simone Meini, Andrea Zanichelli, Rodolfo Sbrojavacca, Federico Iuri, Anna Teresa Roberts, Chiara Suffritti, Carlo Tascini

**Affiliations:** ^1^Internal Medicine Unit, Azienda USL Toscana Centro, Santa Maria Annunziata Hospital, Florence, Italy; ^2^General Medicine Unit, ASST Fatebenefratelli Sacco, Ospedale Luigi Sacco-Università degli Studi di Milano, Milan, Italy; ^3^Infectious Diseases Clinic, Santa Maria Misericordia Hospital, Università degli Studi di Udine, Udine, Italy; ^4^Department of Emergency, Santa Maria Misericordia Hospital, Università degli Studi di Udine, Udine, Italy; ^5^Medical Department, Azienda USL Toscana Nord-Ovest, Pisa, Italy

**Keywords:** SARS-CoV-2, COVID-19, pathophysiology, Contact System, bradykinin, ACE, coagulation

## Abstract

To date the pathophysiology of COVID-19 remains unclear: this represents a factor determining the current lack of effective treatments. In this paper, we hypothesized a complex host response to SARS-CoV-2, with the Contact System (CS) playing a pivotal role in innate immune response. CS is linked with different proteolytic defense systems operating in human vasculature: the Kallikrein–Kinin (KKS), the Coagulation/Fibrinolysis and the Renin–Angiotensin (RAS) Systems. We investigated the role of the mediators involved. CS consists of Factor XII (FXII) and plasma prekallikrein (complexed to high-molecular-weight kininogen-HK). Autoactivation of FXII by contact with SARS-CoV-2 could lead to activation of intrinsic coagulation, with fibrin formation (microthrombosis), and fibrinolysis, resulting in increased D-dimer levels. Activation of kallikrein by activated FXII leads to production of bradykinin (BK) from HK. BK binds to B2-receptors, mediating vascular permeability, vasodilation and edema. B1-receptors, binding the metabolite [des-Arg^9^]-BK (DABK), are up-regulated during infections and mediate lung inflammatory responses. BK could play a relevant role in COVID-19 as already described for other viral models. Angiotensin-Converting-Enzyme (ACE) 2 displays lung protective effects: it inactivates DABK and converts Angiotensin II (Ang II) into Angiotensin-(1-7) and Angiotensin I into Angiotensin-(1-9). SARS-CoV-2 binds to ACE2 for cell entry, downregulating it: an impaired DABK inactivation could lead to an enhanced activity of B1-receptors, and the accumulation of Ang II, through a negative feedback loop, may result in decreased ACE activity, with consequent increase of BK. Therapies targeting the CS, the KKS and action of BK could be effective for the treatment of COVID-19.

## Introduction

Starting in December 2019 in Wuhan (Hubei Province, China), a novel coronavirus, designated SARS-CoV-2, has caused an international outbreak of a respiratory illness (COVID-19), rapidly evolving into a pandemic. The clinical spectrum of SARS-CoV-2 infection varies from asymptomatic or self-limiting mild forms, occurring in most cases, to severe progressive pneumonia with acute respiratory distress syndrome (ARDS), and death. In a yet to be defined percentage of cases, after about one week, there is a sudden and unpredictable worsening of clinical conditions (1).

At present, there is no vaccine or pharmacological treatments of proven efficacy for COVID-19 (2) and further investigation on effective drugs is required to face the current pandemic. One factor determining the lack of effective treatments is that the pathophysiology of COVID-19 remains largely unclear.

In this review, we try to address the complex link between the pathophysiology of COVID-19 and the different proteolytic defense systems operating in human vasculature, investigating the role of the mediators involved and speculating on the possibility of pharmacological modulation.

## Clinical and Laboratory Findings in Patients With COVID-19

COVID-19 is mainly a respiratory illness, but a wide variety of clinical manifestations have been described, including the central nervous (3) and the digestive (4) systems. Most symptomatic COVID-19 patients display manifestations such as fever (98.6%), dry cough (59.4%), dyspnea (31.2%), myalgias (34.8%), sore throat (17.4%), diarrhea (10.1%), and other (5). Olfactory and gustatory dysfunctions are common symptoms, occurring in about 50% of patients and often presenting early in the clinical course (6, 7). Low blood pressure values are frequently observed in hospitalized patients: Wang et al. (5) in their cohort reported a median of mean arterial pressure values of 90 mmHg despite 31% of patients having a history of hypertension.

The predominant findings of lung Computed Tomography are images of bilateral, peripheral and basal ground-glass opacities, crazy-paving pattern, consolidations, often in association (8), and ultrasonography precociously demonstrates a lung interstitial syndrome (9). These findings are consistent with a lung injury characterized by increased permeability, leaky blood vessels and edema, and have been confirmed by the histopathological data obtained from the lungs of patients who died from COVID-19, showing diffuse alveolar damage with necrosis of alveolar lining cells, pneumocyte type 2 hyperplasia, linear intra-alveolar fibrin deposition and increased lung weight due to edema; in addition, thrombi in pulmonary arteries with a diameter of 1–2 mm, without complete luminal obstruction, and massive alveolar capillary microthrombi were observed (10).

Concerning laboratory findings, an increase of lactate dehydrogenase levels and lymphocytopenia are common. Elevated levels of serum ferritin, as commonly found in viral infections, are detected in most patients (11). Levels of interleukin-6 (IL-6) are typically in the upper limit of the reference range and appear to correlate with disease severity (12, 13). IL-6-induced high levels of C-reactive protein (CRP) are typically more related to bacterial rather than to viral infections (11): in COVID-19 patients CRP values are very variable. Even in non-critical patients, high D-dimer levels are found in most patients. Prothrombin time is often slightly increased.

Levels of inflammatory and coagulation biomarkers vary considerably among patients with COVID-19, suggesting the existence of different biochemical/clinical phenotypes, in which the predominant systems involved and the inflammatory and coagulopathy response patterns differ.

From a pathogenetic point of view, it is clear that a link (to date not yet fully clarified) exists between the clinical manifestations and alterations of the inflammatory and coagulation systems, and that these different systems are only apparently unrelated.

## The Role of the Contact System in the Pathophysiology of COVID-19

The Contact System (CS) is part of the innate immune system and of inflammatory response mechanism against artificial material, misfolded and foreign proteins and microorganisms (including viruses), found in the intravascular compartment. It remains to be clarified whether contact factors bind and activate directly on the viral surface or on infected cells (14).

The main proteins of the CS are the Factor XII (FXII), the prekallikrein (PK) and the high-molecular-weight kininogen (HK). These proteins are produced by the liver and circulate as zymogens into the bloodstream. Virtually all plasma PK circulates in complex with HK.

Auto-activation of FXII to FXIIa by contact with a variety of artificial and biological negatively charged surfaces, including microorganisms, gives rise to CS cascade. Biological substances with the potential to support its activation include: DNA, RNA, polyphosphates retained on activated platelet surface, aggregated proteins, neutrophil extracellular traps (NETs) and ferritin (15–19). Kannemeier et al. (20) presented evidence that different forms of eukaryotic and prokaryotic RNA serve as promoters of blood coagulation, enhancing auto-activation of proteases of the CS, such as FXII and FXI. As the extracellular RNA derived from damaged or necrotic cells represented a “foreign surface” able to activate the CS, it could be speculated that the same process may be initiated by viral RNA. In addition, at times of cellular stress (i.e., hypoxia, hyperthermia, oxygen radical production) such as that observed during COVID-19, endogenous “alarmins” named “Danger-Associated Molecular Patterns” (DAMPs) are released from necrotic cells. These molecules are able to initiate appropriate defense reactions associated with “sterile” inflammation and tissue repair, engaging the “Pattern-Recognition Receptors” (PRRs), such as the cell membrane and endosomal Toll-like receptors (TLRs) (21). Moreover, during viral infections, TLRs represent a host primary line of defense for pathogen sensing, due to their properties to bind diverse exogenous ligands (the “Pathogen-Associated Molecular Patterns,” PAMPs), including viral RNA (21); DAMPs and PAMPs are able to activate the FXII and the CS.

HK, complexed with PK, binds to these “surfaces”: the domain 5 is the artificial surface-binding region of HK, while the domain 6 binds PK and FXI in order to initiate the intrinsic coagulation (16). After HK binds to a surface, PK is exposed to conversion to plasma kallikrein (KAL) by FXIIa: the binding induces a conformational change in PK so that it acquires enzymatic activity and can stoichiometrically cleave HK (22). In turn, KAL cleaves and activates more FXII, in a powerful positive feedback loop (14).

In addition, a vessel wall-associated serine protease, prolyl-carboxypeptidase (PRCP), is able to activate PK to KAL independent of FXIIa (16).

The CS is involved in inflammation and in coagulation: when sufficient amounts of FXII are activated, FXIIa also activates FXI (to FXIa), and the intrinsic (or contact) coagulation pathway can start, leading to subsequent thrombin activation and fibrin formation. KAL can influence the fibrinolytic pathway by activating plasminogen into plasmin, thus leading also to fibrin degradation (23). D-dimer is a soluble fibrin degradation product deriving from the plasmin-mediated degradation of cross-linked fibrin: it can therefore be considered a biomarker of concomitant activation of both coagulation and fibrinolysis (24).

It should be remembered that plasmin can also activate FXII (25), and that FXIIa can act as a plasminogen activator too (26): it has been speculated that in the very early stages of *in vivo* contact activation, when PK has yet to become activated, plasmin could have an initiating role (26), however it should be noted that plasmin is hardly present in plasma as an active protease due to the very effective action of its specific inhibitor, the α2-anti-plasmin: plasmin is protected from inactivation by this inhibitor only when bound to fibrin.

The coagulation cascade can be modernly considered as a component and one of the intravascular effectors of innate immunity (immunothrombosis) (27). It is debatable whether the main physiological function of FXII is the activation of the intrinsic coagulation pathway, or if to be a component of the CS should be considered its main physiological function. For physiological hemostasis to occur, FXII auto-activation is dispensable (21). The FXII-induced intrinsic coagulation pathway is involved in pathological thrombus formation but is not associated with abnormal hemostasis: FXII-deficient subjects present in fact a normal hemostatic capacity (28, 29). Challenging the concept of the coagulation balance, targeting FXII or its activator polyphosphate can provide protection from thromboembolic diseases (and modulate immunothrombosis) without interfering with hemostasis and increasing the risk of bleeding (14, 16, 18, 30, 31).

COVID-19 is a condition clearly characterized by coagulopathy, as testified by the extensive microthrombosis reported in lung autopsies (10), and the high levels of D-dimer displayed by most patients indirectly testify the hyperactivation of both coagulation and fibrinolysis, and overwhelming immunothrombosis. It should be remembered that low-molecular weight heparins (LMWHs) have been extensively used in hospitalized COVID-19 patients for preventing venous thromboembolism and thrombotic complications, and are currently investigated in randomized controlled trials (i.e., ClinicalTrials.gov Identifier: NCT04401293).

It is interesting to note that in the physiological state FXII acts as a growth factor promoting angiogenesis and wound repair (32), but pathologically it can promote lung fibroblast proliferation leading to pulmonary fibrosis (33): COVID-19 may also evolve into pulmonary fibrosis.

The archetypal contact activation disease state is sepsis from any etiology. There is no specific data on the model of SARS-CoV-2, but data may be gathered from other viral models. It is known that herpes simplex virus type-1 (HSV1) can trigger and amplify coagulation through the contact phase and intrinsic coagulation pathway: both an inhibitor of FXIIa (corn trypsin inhibitor), and anti-FXII, anti-KAL and anti-FXI antibodies were able to inhibit HSV1-initiated clotting (34). Moreover, PK and FXII levels are significantly lower in patients with dengue hemorrhagic fever (DHF), probably due to activation and consumption (35).

It has been mentioned that CS is part of the innate immune system: it is known that non-structural protein 3 (nsp3) of coronaviruses results able to block the host innate immune response (36), and other nsp play a role in evading host recognition (37).

## The Kallikrein–Kinin System

The Kallikrein–Kinin System (KKS) is mainly a host inflammatory response mechanism, and although KKS and CS overlap and interact in the intravascular compartment (plasma KAL is part of both systems), the use of the two terms has different implications. Activation of KKS finally leads to the liberation of bradykinin (BK), and plays an essential role in inflammation, but not in blood coagulation (16).

Upon activation by FXIIa, KAL cleaves HK, releasing from its domain 4 the nonapeptide bradykinin (BK-1-9 or BK) (38); BK is converted by a carboxypeptidase to [des-Arg^9^]-BK (BK-1-8 or DABK), an active metabolite (39). During inflammation, plasmin potentiates the cleavage of HK by KAL, thus enhancing BK production (40).

BK and DABK bind to two pharmacologically distinct G protein-coupled receptors: the bradykinin B2 receptor (B2R), whose ligand is BK, and the B1 receptor (B1R), whose main agonist is DABK (39). The B2R is widely and constitutively expressed in mammalian cells (e.g., endothelial and smooth muscle cells), whereas the B1R is mostly inducible under the effect of cytokines during infections and immunopathology (41).

After binding through its B2R, BK activates signaling pathways resulting in increased vascular permeability, vasodilation, edema formation, hypotension, pain, fever (14): all typical clinical features of COVID-19. BK is one of the most potent vasodilatory substances in humans: it is known that the BK-mediated angioedema is responsible for a very high percentage of serious morbidity and mortality (42). BK is also one of the most potent inflammatory mediators, able to stimulate the production of superoxide radicals and nitric oxide and to modulate the mobilization and release of histamine, arachidonic acid, prostaglandin E2, prostacyclin, pro-inflammatory interleukin-1, and tumor necrosis factor (TNF)-alpha (41). Thereafter, BK has shown to increase IL-6 production via B2R in colorectal cancer cell (43), and the B2R-antagonist icatibant was able to inhibit the BK-induced IL-6 release (44). This effect is interesting: also chloroquine, that has been extensively used and investigated for COVID-19 treatment, was able to reduce IL-6 production by monocytes/macrophages (45). BK also stimulates tissue plasminogen activator (t-PA) release from human endothelium through a B2R-dependent mechanism: this effect was significantly reduced in smokers (46). A strong link between KKS and the renin–angiotensin system (RAS) is testified by the fact that B2R forms homo- and heterodimers with several receptors of the RAS, that are important for some physiologic functions, including thrombosis risk regulation. The B2R also complexes with endothelial cell nitric oxide synthase, while the B1R couples with inducible nitric oxide synthase (16).

B1R mediates several responses including vasodilation, hypotension, and increased vascular permeability (41): all typical features of COVID-19.

Human kallikreins have been detected in many tissues (47), including the epithelia of the upper and lower respiratory tract: there are in fact two classical pathways for the generation of kinins, the plasma and the tissue KKS. As the substrate of plasma KAL is HK (leading to BK), the substrate of tissue kallikreins is the low-molecular-weight kininogen, leading to formation of the decapeptide Lys-bradykinin or kallidin (KD). A carboxypeptidase leads to the formation of the active metabolite [des-Arg^10^]-KD (DAKD) from KD. KD mainly binds to B2R, while B1R has a high affinity for DAKD (48).

It is not known if SARS-CoV-2 infection is specifically associated with kinins dysregulation, but this happens in several viral models. Low levels of HK have been observed in DHF patients, perhaps due to proteolysis and generation of BK (49). Taylor et al. (50) previously described a novel mechanism of hantavirus-induced vascular leakage involving activation of the KKS, showing that incubation of FXII, PK, and HK with hantavirus-infected endothelial cells results in increased cleavage of HK, higher enzymatic activities of FXII/KAL and increased liberation of BK, that dramatically increased cell permeability. Furthermore, the alterations in permeability could be prevented using inhibitors directly blocking BK binding, the activity of FXII, or the activity of KAL (50). Infection of guinea pigs by nasal instillation of parainfluenza-3 virus induced airway hyperreactivity and influx of inflammatory cells into lung tissues, and these responses were attenuated by B2R-antagonists (51). Tissue kallikrein 1 was shown to intervene early during influenza infection, enhancing the antiviral defense, and the decreased expression observed in patients with chronic obstructive pulmonary disease could contribute to the less favorable evolution of influenza in this group (52).

Therefore, the KKS appears to be involved in vascular leakage and inflammatory response observed during different viral infections (14). We can speculate that modulation of the CS and the KKS may limit the evolution towards a frankly dysregulated host response also in SARS-CoV-2 infection.

Moreover, a role of BK in COVID-19 pathogenesis is suggested by several clinical features and symptoms observed in patients: given the close interconnection with the RAS, these aspects will be further discussed in the next chapter.

## The Renin–Angiotensin System (RAS) and the Interplay With KKS

The renin–angiotensin system (RAS) is classically known for its effects on the cardiovascular system and fluid homeostasis, but it has become clear that the RAS is present in many tissues, where evidently has a role to play (53).

Starting from angiotensinogen, whose primary source is the liver, the RAS leads to the production of the multi-functional peptide hormone Angiotensin II (Ang II). Renin first catalyzes the cleavage of the peptide Angiotensin I (Ang I) from the N-terminus of the angiotensinogen molecule, then, sequentially, the dicarboxyl-peptidase angiotensin converting enzyme (ACE) removes two amino-acids from the C-terminus of Ang I to form Ang II (54). Ang II exerts its main functions binding to two specific G-protein coupled receptors: the ATII type 1 receptor (AT1R) and ATII type 2 receptor (AT2R) (54).

ACE is present in many tissues and is particularly abundant on the endothelium of the lungs: it is mainly anchored to the plasma membrane through a single trans-membrane domain, but a soluble form has also been described (53).

Apart from its well-known role as a peptidyl-dipeptidase forming Ang II, ACE is also described as a kininase II, able to inactivate BK, as well as KD (53). The affinity of ACE appears to be higher for BK than for Ang I, suggesting that ACE-inhibition may really involve the BK degradation more than the Ang II production (55). BK-evoked sensitization of airway sensory nerves is believed to be the main mechanism for ACE-inhibitor-induced dry cough (56): considering that dry cough is very frequently observed in COVID-19 patients, this pathway could in part explain the pathogenesis of this symptom. Additionally, a role of the BK has been hypothesized also for gustatory and olfactory dysfunctions (7); again, ACE-inhibitors can cause olfactory dysfunction (57).

In addition, over the last 20 years, knowledge of the biology and physiology of another enzyme besides ACE, the angiotensin converting enzyme 2 (ACE2), has accumulated (58): ACE2 is widely expressed, including type 2 alveolar epithelial cells, endothelial cells and enterocytes (10, 58).

Both ACE and ACE2 act as zinc metallopeptidases (ACE2 only acts as a carboxypeptidase), but differ for substrate specificities, displaying counterbalancing roles in the RAS.

ACE2 converts Ang I into Angiotensin (1-9), and Ang II into Angiotensin (1-7); unlike ACE, ACE2 does not cleave BK, and is insensitive to conventional ACE-inhibitors (58). Ang II can be converted to angiotensin (1-7) also by PRCP in the low-pH areas of the kidney (59).

Angiotensin 1-7, acting on Mas receptor, exerts vasodilatory effects, thus diminishing and opposing the vasoconstrictor effect, mainly AT1R-mediated, of Ang II; moreover, it displays anti-fibrotic, anti-oxidant and anti-hypertrophic protective properties (58).

Therefore, ACE2 expression seems to protect from lung injury. Sodhi et al. (39) observed that a reduction in pulmonary ACE2 activity contributes to the pathogenesis of lung inflammation, resulting in prompt onset of neutrophil infiltration and more severe inflammation. Imai et al. (60) showed that the loss of ACE2 expression in acute lung injury leads to leaky pulmonary blood vessels through AT1R stimulation, while the AT2R protects against lung injury during sepsis. Angiotensin 1-9 has shown beneficial biological effects via the AT2R, resulting in protective effects on cardiac and vascular remodeling (58) and against pulmonary arterial hypertension, inflammation and fibrosis (61).

SARS-CoV-2 binds ACE2 for host cell entry, through the binding of its major spike glycoprotein (S1) to the N-terminal region of the receptor (62); chloroquine seems to interfere with ACE2 glycosylation, thus possibly preventing SARS-CoV-2 binding to target cells (63). Following binding with SARS-CoV-2, a loss of ACE2 function occurs, driven by endocytosis and activation of proteolytic cleavage and processing (58, 62). It can be assumed that this downregulation may be involved in the pathophysiology of COVID-19 and its manifestations. DABK is a substrate of ACE2, and the attenuation of ACE2 activity leads to impaired DABK inactivation and thus to enhanced B1R signaling.

In a mouse model, the lack of ACE2 function with consequent accumulation of Ang II, through a negative feedback loop, resulted in a secondary reduction of ACE activity (at the molecular level, Ang II downregulates renal ACE gene and enzymatic activity levels, as well as renin gene expression): these crosstalk effects between ACE2 and ACE appeared to be sex-dependent and more evident in males (64). It is known that COVID-19 affects male patients in a larger percentage (65) and with worse outcomes (66). The reduced activity of ACE is also expected to result in further BK accumulation. Moreover, it has been recognized *in vitro* that Ang II, through the stimulation of AT2R, is associated with increased expression of PRCP, leading to a KAL-mediated increased formation of BK (67).

Since SARS-CoV-2 binds to ACE2 receptors to enter host cells, and intravenous infusion of ACE-inhibitors and angiotensin receptor blockers (ARBs) in experimental animal models increased the amount of ACE2 receptors in the cardiopulmonary circulation, it has been speculated that patients chronically taking these drugs may be at increased risk of worse outcomes from COVID-19 (68). However, to date, there are no conclusive data demonstrating beneficial or adverse outcomes with background use of ACE-inhibitors, ARBs or other RAS antagonists among COVID-19 patients with a history of cardiovascular disease treated with these drugs (69–71). For the pathophysiological considerations previously made, however, in our opinion, it remains debatable if ACE-inhibitors, for their action on BK, should be temporarily suspended during the acute phase of illness, especially in the case of low blood pressure values.

Finally, it is interesting to observe that in an experimental malaria model (*Plasmodium* parasites during blood stages release kinins), exposure to captopril (an ACE-inhibitor that leads to the reduction of BK degradation) resulted in death in mice, while the concomitant administration of chloroquine protected them. B1R-knockout mice presented a significant reduction of survival when compared with wild-type mice, unlike the B2R-knockout ones (72). In this inflammation/infection model, chloroquine-induced upregulation of B1R expression proved protective: the full meaning of this result is unclear but might indicate that the selective inhibition of B2R could represent a rational modulation of dysregulated BK pathway during infection. Could the same considerations apply to COVID-19?

## C1-Inhibitor and Its Potential Role in Viral Infections

Hereditary angioedema (HAE) represents the archetypal KKS disorder and can be due to a deficiency of C1-INH (Type 1), an abnormal C1-INH molecule (Type 2), or a gain-in-function of FXII with consequent plasma C1-INH consumption (Type 3) (73). Thrombin formation is not considered a feature of this disorder: even if patients with acute attacks have elevated D-dimer levels, they do not display an increased thrombotic risk (74). Clinical pictures of activation of CS and KKS without (such as HAE) and with thrombin formation (such as sepsis) can be in fact distinguished (16): COVID-19 evidently falls into the latter group.

C1-INH is a protein able to inhibit multiple serine proteases involved in the CS, KKS, Complement, Fibrinolysis, and Coagulation Systems: through the inhibition of C1r and C1s subcomponents of C1 complex, FXIIa, and KAL, C1-INH prevents the activation of CS and KKS. The N-terminal end (non-serpin domain) confers to C1-INH the capacity to bind lipopolysaccharides and E-selectin: owing to this moiety, C1-INH can also intervene in the regulation of inflammatory reactions (75). Moreover, C1-INH inhibits selectin-mediated leukocyte adhesion, regardless of its protease inhibitory activity (76).

Wygrecka et al. (77) showed that C1-INH is able to inhibit the cytotoxic activity of extracellular histones (that play a determining role in pulmonary injury leading to ARDS) and the release of several cytokines, such as TNF-alpha, IL-1b, and IL-6. It is interesting to note that accumulation of extracellular histones has been detected during infection due to influenza virus, and anti-histone antibodies have led to a marked decrease in the lung damage consisting of widespread pulmonary microvascular thrombosis, endothelial necrosis, hemorrhagic effusions and edema (78). These histopathological findings are observed also in COVID-19, although there are several differences compared to the influenza model (10) whose discussion goes beyond the scope of this review. Although there is actually no specific evidence regarding SARS-CoV-2 infection, it can be assumed that C1-INH might have beneficial effects also in this case, both through the inhibition of the CS and KKS, especially regarding the BK-induced vascular leakage and edema formation, and its anti-inflammatory activity mediated by inhibition of complement activation and histone toxicity.

**FIGURE 1:**
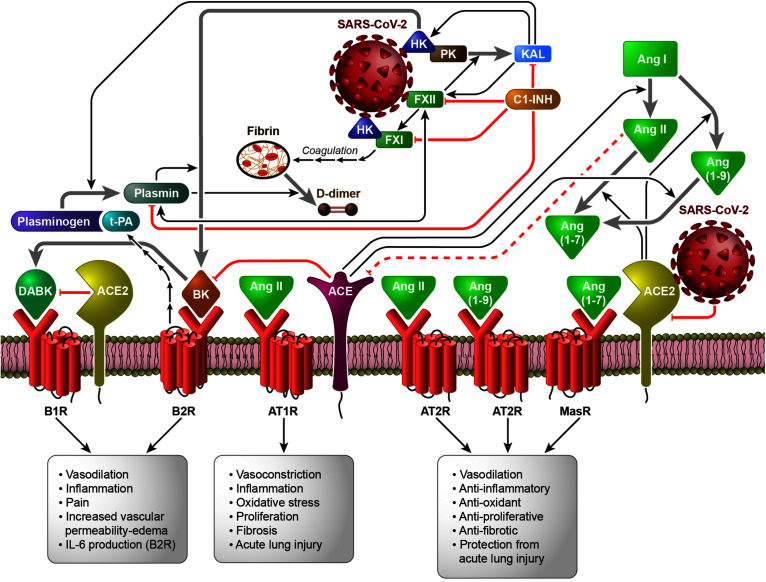
The Contact System (CS) as a plausible link between Coagulation/Fibrinolysis, Kallikrein–Kinin and Renin–Angiotensin Systems in the pathobiology of SARS-CoV-2 infection (based on the hypothesis of the activation of FXII and HK by SARS-CoV-2, directly or following cell invasion and/or damage). Black arrows indicate enzymatic activation, while red lines represent inhibition or degradation/downregulation. The sequences of black arrows imply the involvement of other molecules not shown to activate the molecule indicated by the final arrow. Dashed red lines imply the involvement of other molecules not shown to inhibit the molecule indicated at the end of the line. Gray arrows indicate the transformation of one molecule into another. *ACE*, angiotensin converting enzyme; *ACE2*, angiotensin converting enzyme 2; *Ang I*, Angiotensin I; *Ang II*, Angiotensin II; *Ang* (1-7), Angiotensin 1-7; *Ang* (1-9), Angiotensin 1-9; *AT1R*, ATII type 1 receptor; *AT2R*, ATII type 2 receptor; *B1R*, B1 receptor; *B2R*, B2 receptor; *BK*, bradykinin; *C1-INH*, C1-inhibitor; *DABK*, [des-Arg^9^]-BK; *FXI*, coagulation factor XI; *FXII*, coagulation factor XII; *HK*, high-molecular-weight kininogen; *IL-6*, interleukin-6; *KAL*, plasma kallikrein; *MasR*, Mas receptor; *PK*, plasma prekallikrein; *t-PA*, tissue plasminogen activator.

Figure 1 shows the interconnection between the different human proteolytic systems operating in the vasculature, proposing a picture of an integrated host response to SARS-CoV-2 infection.

Table 1 lists some available drugs potentially representing effective therapeutic approaches in COVID-19, by modulation of the pathways and systems whose involvement has been hypothesized in its pathogenesis.

**TABLE 1:** Potential therapeutic approaches able to modulate the systems involved in the pathogenesis of COVID-19.

**Drug**	**Mechanism of action**	**Labeled indication**	**Potential role in COVID-19**
**Contact system**			
C1-inhibitor	Inhibition of CS, Coagulation/Fibrinolytic systems, complement and KKS	Treatment and prevention of angioedema attacks in hereditary angioedema	Inhibition of all systems involved
Anti-factor XII (FXII) antibody	Monoclonal antibody inhibiting FXII	Phase II study ongoing; phase III study planned. Studied indication: prevention of angioedema attacks	Inhibition of FXII and consequently of CS and KKS
			Prevention and treatment of thrombosis, without increasing bleeding risk (Action on Coagulation system)
**Kallikrein–kinin system**			
Icatibant	Bradykinin type 2 receptor (B2R)-antagonist	Treatment of acute attacks in hereditary angioedema	Inhibition of pro-inflammatory and vasoactive actions of BK
Lanadelumab	Monoclonal antibody inhibiting plasma KAL	Prevention of attacks of hereditary angioedema	Inhibition of KKS and BK generation
Ecallantide	Inhibition of plasma KAL	Treatment of acute attacks in hereditary angioedema	Inhibition of KKS and BK generation
**Coagulation system**			
Low-Molecular-Weight Heparin/Fondaparinux	Catalyzed inhibition of activated coagulation factor X by antithrombin	Prevention and treatment of venous thromboembolism	Prevention and treatment of thrombosis and pulmonary embolism (consider bleeding risk)
Anti-factor XII (FXII) antibody	See above	See above	See above

## Discussion and Conclusion

The hypothesis of the involvement of different human proteolytic defense systems operating in the vasculature in the pathogenesis of COVID-19 has recently been proposed also by other authors. van de Veerdonk et al. (79) hypothesized that a kinin-dependent local lung angioedema via B1R and eventually B2R is an important feature of COVID-19 and proposed that blocking the B2R and inhibiting plasma KAL activity might be beneficial in early disease, preventing ARDS. Roche and Roche (80) emphasized the pivotal role of BK and DABK, suggesting that the B2R-antagonist icatibant might be able to interrupt the dysregulated pathway, thereby improving clinical outcomes. Colarusso et al. (23) proposed instead to block pharmacologically the KKS upstream of the BK, by means of lanadelumab. Regarding B1R-antagonists, several companies have in past developed orally available molecules, and some of these entered phase II clinical trials, but none have been developed further; possible reasons for this failure may be inefficacy in humans due to species differences, or human specific adverse effects (48).

In our opinion, the rational for modulating these pathways is strong but to date few data for COVID-19 are available. However, the exceptional nature of this pandemic and the lack of effective interventions of proven efficacy makes it necessary to explore further therapeutic possibilities.

Understanding the pathogenetic mechanisms underlying COVID-19 is crucial for the development of new effective therapeutic approaches modulating the CS, the KKS, the RAS and the Coagulation/Fibrinolysis System. The KKS inhibitors lanadelumab and ecallantide, licensed for the treatment of HAE, and several oral KKS inhibitors in clinical development, should be assessed for their efficacy in the treatment of patients with COVID-19. The same holds for icatibant, a selective B2R antagonist used for on demand treatment in HAE. Other promising CS-linked targets or mediators that should be explored in COVID-19 include anti-FXIIa antibodies and C1-INH. This pathophysiological therapeutic approach could be of great value also for other viral infections.

## Data Availability Statement

Publicly available datasets were analyzed in this study. This data can be found at the appropriate doi link of every cited article.

## Author Contributions

SM, AZ, RS, FI, and CT: conceptualization. SM, AZ, RS, FI, CS, AR, and CT: formal analysis. AZ: funding acquisition. SM, AZ, and CT: investigation and project administration. SM, AZ, RS, FI, AR, CS, and CT: methodology and resources. SM, AZ, AR, and CT: writing – original draft preparation and writing – review and editing. All authors contributed to the article and approved the submitted version.

## Conflict of Interest

AZ received speaker/consultancy fees and/or was a member of medical/advisory boards for CSL Behring, Shire/Takeda, and SOBI. All the authors declare that they have no financial competing interests about the topic of this article, except for the publishing support and journal styling services, that were provided by SEEd Medical Publishers and funded by CSL Behring, Italy.

## Abbreviations

ACE: Angiotensin-Converting Enzyme
ACE2: Angiotensin-Converting Enzyme 2
Ang I: Angiotensin I
Ang II: Angiotensin II
Ang (1-7): Angiotensin 1-7
Ang (1-9): Angiotensin 1-9
ARB: angiotensin receptor blocker
ARDS: Acute respiratory distress syndrome
AT1R: Angiotensin II type 1 receptor
AT2R: Angiotensin II type 2 receptor
BK: Bradykinin
B1R: Bradykinin B1 receptor
B2R: Bradykinin B2 receptor
COVID-19: COronaVIrus Disease 2019
CS: Contact System
C1-INH: C1-inhibitor
DABK: [des-Arg^9^]-Bradykinin
DAKD: [des-Arg^10^]-Kallidin
DAMPs: Danger-Associated Molecular Patterns
DHF: dengue hemorrhagic fever
FXI: Factor XI
FXII: Factor XII
HK: High-molecular-weight Kininogen
HSV1: Herpes simplex virus type-1
IL-6: Interleukin-6
KAL: Kallikrein
KD: Kallidin
KKS: Kallikrein–Kinin System
LMWH: Low-Molecular Weight Heparin
PAMPs: Pathogen-Associated Molecular Patterns
PK: Prekallikrein
PRCP: Prolyl-carboxypeptidase
PRR: Pattern-Recognition Receptor
RAS: Renin–Angiotensin System
SARS-CoV-2: Severe Acute Respiratory Syndrome – Coronavirus – 2
TLR: Toll-like receptor
TNF-alpha: tumor necrosis factor-alpha
t-PA: tissue-Plasminogen Activator.
